# A gut bacterial signature in blood and liver tissue characterizes cirrhosis and hepatocellular carcinoma

**DOI:** 10.1097/HC9.0000000000000182

**Published:** 2023-06-14

**Authors:** Maria Effenberger, Silvio Waschina, Christina Bronowski, Gregor Sturm, Oronzo Tassiello, Felix Sommer, Andreas Zollner, Christina Watschinger, Felix Grabherr, Ronald Gstir, Christoph Grander, Barbara Enrich, Reto Bale, Daniel Putzer, Angela Djanani, Alexander R. Moschen, Heinz Zoller, Jan Rupp, Stefan Schreiber, Remy Burcelin, Cornelia Lass-Flörl, Zlatko Trajanoski, Georg Oberhuber, Philip Rosenstiel, Timon E. Adolph, Konrad Aden, Herbert Tilg

**Affiliations:** 1 Department of Internal Medicine I, Gastroenterology, Hepatology, Endocrinology & Metabolism, Medical University of Innsbruck, Innsbruck, Austria; 2 Institute for Human Nutrition and Food Science, Division of Nutriinformatics, Christian-Albrechts-University of Kiel, Kiel, Germany; 3 Institute of Clinical Molecular Biology, Christian-Albrechts-University and University Hospital Schleswig-Holstein, Campus Kiel, 24105 Kiel, Germany; 4 Biocenter, Institute of Bioinformatics, Medical University of Innsbruck, Innsbruck, Austria; 5 Department of Internal Medicine I, Gastroenterology, Nephrology, Metabolism & Endocrinology, Johannes Kepler University, Linz, Austria; 6 Institute of Hygiene and Medical Microbiology, ECMM, Medical University of Innsbruck, Innsbruck, Austria; 7 Department of Radiology, Medical University of Innsbruck, Innsbruck, Austria; 8 Christian Doppler Laboratory for Mucosal Immunology, Johannes Kepler University, Linz, Austria; 9 Department of Infectious Diseases and Microbiology, University Hospital Schleswig-Holstein, Luebeck, Germany; 10 Department of Internal Medicine I, Christian-Albrechts-University and University Hospital Schleswig-Holstein, Campus Kiel, Kiel, Germany; 11 INSERM 1297 and University Paul Sabatier: Institut des Maladies Métaboliques et Cardiovasculaires, France and Université Paul Sabatier, Toulouse, France; 12 INNPATH, Institute of Pathology, University Hospital of Innsbruck, Innsbruck, Austria

## Abstract

HCC is the leading cause of cancer in chronic liver disease. A growing body of experimental mouse models supports the notion that gut-resident and liver-resident microbes control hepatic immune responses and, thereby, crucially contribute to liver tumorigenesis. However, a comprehensive characterization of the intestinal microbiome in fueling the transition from chronic liver disease to HCC in humans is currently missing.

**Methods:**

Here, we profiled the fecal, blood, and liver tissue microbiome of patients with HCC by 16S rRNA sequencing and compared profiles to nonmalignant cirrhotic and noncirrhotic NAFLD patients.

**Results:**

We report a distinct bacterial profile, defined from 16S rRNA gene sequences, with reduced α-and β-diversity in the feces of patients with HCC and cirrhosis compared to NAFLD. Patients with HCC and cirrhosis exhibited an increased proportion of fecal bacterial gene signatures in the blood and liver compared to NAFLD. Differential analysis of the relative abundance of bacterial genera identified an increased abundance of Ruminococcaceae and Bacteroidaceae in blood and liver tissue from both HCC and cirrhosis patients compared to NAFLD. Fecal samples from cirrhosis and HCC patients both showed a reduced abundance for several taxa, including short-chain fatty acid–producing genera, such as *Blautia* and *Agathobacter.* Using paired 16S rRNA and transcriptome sequencing, we identified a direct association between gut bacterial genus abundance and host transcriptome response within the liver tissue.

**Conclusions:**

Our study indicates perturbations of the intestinal and liver-resident microbiome as a critical determinant of patients with cirrhosis and HCC.

## INTRODUCTION

HCC is an aggressive malignancy, which almost exclusively arises in patients with chronic liver disease (CLD). Worldwide, it is the third leading cause of cancer mortality and shows growing incidence.^1^ Between 1975 and 2005, the age-adjusted incidence of HCC rose from 1.6 to 4.5 per 100,000 people in the United States.^2^ HCC is responsible for more than 700,000 deaths annually.^3^ Despite improvements in early recognition by surveillance programs and development in pharmacotherapies, the one-year survival for HCC is still less than 50%.^3,4^ While viral hepatitis, especially chronic HBV and chronic HCV infection, is the leading cause of cirrhosis and HCC in low-income countries and Eastern Asia, alcohol-associated liver disease and NAFLD are mainly causative for cirrhosis and HCC in high-income countries.^5–7^ The pathogenesis of HCC is versatile, driven by a circle of liver injury, inflammation, and regeneration that typically span decades.

Increasing evidence points toward the bacterial microbiome as a key player in health and disease,^8–10^ and it was also noted that dysbiosis of the gut microbiota may contribute to carcinogenesis in other remote organs.^11–13^ Dysbiosis is thought to be a consecutive downstream event resulting from an inflammatory state driven by the underlying liver disease, leading to a vicious cycle of further dysbiosis-driven inflammation and disrupted host-microbial crosstalk. Profound disturbance in the intestinal microbiome has been shown to promote carcinogenesis not only in the gastrointestinal tract, such as colorectal carcinoma,^14,15^ but also fuels carcinogenesis at remote and systemic body compartments, such as melanoma, leukemia, or bronchial carcinoma.^16–18^ Furthermore, certain microbial patterns in the blood and tissue of cancer patients are attributed to different cancer types.^19^

Increased translocation of the intestinal microbiome is common in patients with CLD and causes multiple complications in advanced disease stages.^20^ Dysbiosis of the bacterial microbiome seems to be a key player in promoting liver diseases and the subsequent development of HCC.^21–23^ The liver does not only receive nutrients-rich blood from the intestine, but it is also the first target of the intestinal microbiota and microbe-associated molecular patterns, which can elicit inflammatory responses via pattern-recognition receptors and microbial metabolites.^24^ The multilayer intestinal barrier ensures that hepatic exposure to pro-inflammatory microbe-associated molecular patterns is minimal. However, a failing gut barrier due to alterations of the gut microbiota in CLD may contribute to chronic inflammation and the progression of liver diseases, which increases the risk of HCC development as the final stage of the disease process.^25–29^ Although previous studies have identified substantial changes in the fecal microbiome of HCC patients, little is known about liver-specific changes in the microbiome or at least in part of the corresponding bacterial DNA signature, which could correspond to live or dead bacteria, both with an inflammatory potential. Here, we set out to systematically analyze the fecal, blood, and liver microbiome in NAFLD, cirrhosis, and HCC patients to generate a microbiota disease trajectory toward HCC development across various body compartments. We, thereby, find that HCC patients present with altered microbial diversity and increased translocation of fecal 16S rRNA bacterial components into the bloodstream and liver. Most notably, we do not find HCC-specific changes in the tissue-resident microbial composition defined from 16S rRNA gene sequences but rather identify disturbances in the liver-specific interaction between microbiota and genes, which are uniquely differentially expressed in HCC samples. These data indicate that the microbiota might affect HCC pathogenesis through a misled tissue-specific host–microbial interaction.

## METHODS

### Patients

The diagnosis of HCC was established by either abdominal CT or abdominal MRI. HCC was diagnosed with either multiphase CT or multiphase MRI with a Liver Reporting and Data System Score of 5 (AASLD and EASL). The patient’s characteristics of all patients are listed in Table 1, and the patient’s characteristics undergoing liver biopsy are listed in Table 2.

**TABLE 1:** Patient’s characteristics overall

	NAFLD (n = 21)	Compensated cirrhosis (n = 27)	HCC (n = 111)		*p*		
Age (y)	58 (±17.2)	61 (±11.2)	67.5 (±20.3)		G1/G2	G1/G3	G2/G3
	—	—	—		NS	0.02	NS
Sex (female %)	10 (47.6)	8 (29)	39 (35)		NS		
Weight (kg)	84.3 (±22.4)	72.1 (±18.4)	76.3 (±11.4)		G1/G2	G1/G3	G2/G3
	—	—	—		0.03	0.01	NS
Height (cm)	173.3 (±9.2)	172.9 (±10.4)	173.1 (±8.0)		NS		
BMI (kg/m2)	30.1 (±4.7)	26.4 (±5.2)	26.3 (±3.3)		G1/G2	G1/G3	G2/G3
	—	—	—		0.02	0.02	NS
AFLD	NA	12	54		NS		
NAFLD	21	10	36		G1/G2	G1/G3	G2/G3
	—	—	—		0.001	0.001	NS
Hepatitis B	NA	NA	1		NA		
Hepatitis C	NA	5	9		NS		
PBC/PSC/AIH	NA	NA	11		NA		
MELD	NA	9.7 (±4.9)	8.5 (±7.2)		NS		
BCLC staging	NA	NA	0	32	NA		
	—	—	A	48	—		
	—	—	B	26	—		
	—	—	C	5	—		
Leucocytes (g/l)	3.8 (±2.1)	4.2 (±2.7)	4.8 (±3.1)		NS		
Hemoglobin (g/l)	142.3 (±17.2)	129.0 (±26.3)	131.8 (±22.1)		NS		
Hematocrit (%)	0.42 (±0.06)	0.37 (±0.07)	0.38 (±0.05)		NS		
Platelet count (g/l)	321.4 (±68.3)	267.9 (±82.6)	291.3 (±67.9)		G1/G2	G1/G3	G2/G3
	—	—	—		0.002	0.006	NS
Urea (mg/dl)	34.2 (±17.8)	36.7 (±16.9)	35.9 (±19.6)		NS		
Creatinine (mg/dl)	0.6 (±0.2)	1.0 (±0.5)	0.8 (±0.9)		NS		
Filtration rate (ml/min/m2)	59.3 (± 10.2)	58.2 (± 11.4)	59.0 (± 13.8)		NS		
Bilirubin (mg/dl)	0.6 (±0.5)	1.1 (±0.9)	0.9 (±1.2)		NS		
Sodium (mmol/l)	141.2 (±3.8)	139.2 (±5.1)	142.1 (±6.2)		NS		
AST (U/l)	41.6 (±14.8)	58.5 (±33.6)	61.2 (±51.2)		NS		
ALT (U/l)	31.2 (±11.4)	42.9 (±27.2)	45.3 (±31.3)		NS		
Gamma-GT (U/l)	67.2 (±41.6)	72.4 (±39.3)	70.1 (±51.2)		NS		
Alkaline Phosphatase (U/l)	74.5 (±34.2)	89.6 (±72.7)	92.4 (±88.2)		NS		
LDH (U/l)	201.3 (±61.7)	234.7 (±101.2)	178.2 (±91.5)		NS		
CRP (mg/dl)	0.7 (±0.8)	1.4 (±0.3)	1.2 (±0.7)		NS		
Quick (%)	99.7 (±14.3)	65.5 (±19.7)	81.7 (±17.3)		G1/G2	G1/G3	G2/G3
	—	—	—		0.001	NS	0.003
INR	0.9 (±0.4)	1.4 (±0.3)	0.8 (±0.7)		G1/G2	G1/G3	G2/G3
	—	—	—		0.003	NS	0.005
PT (sec)	33.5 (±3.2)	39.4 (±8.3)	36.5 (±5.6)		G1/G2	G1/G3	G2/G3
	—	—	—		0.01	NS	0.05
Fibrinogen (mg/dl)	342.0 (±57.3)	267.4 (±109.3)	322.3 (±97.4)		NS		
Albumin (mg/dl)	4190.2 (±401.4)	3690.6 (±353.8)	3990.8 (±421.9)		G1/G2	G1/G3	G2/G3
	—	—	—		NS	0.02	NS
Alpha-1-Fetoprotein (µg/l)	2.1 (±0.5)	7.5 (±3.8)	22.5 (±14.5)		G1/G2	G1/G3	G2/G3
	—	—	—		NS	0.002	0.004
Laxative use (%)	4 (19.1)	1 (3.7)	13 (11.7)		NS		
PPI use (%)	5 (23.8)	2 (7.4)	24(21.6)		NS		
Type 1 /Type 2 Diabetes (%)	7 (33)	4 (14.5)	18 (16.2)		G1/G2	G1/G3	G2/G3
	—	—	—		0.003	0.007	NS

Note: Data are expressed as case numbers (%) or mean ± SD.

Abbreviations: µg/l, microgram/liter; AFLD, alcohol-associated fatty liver disease; AIH, autoimmune hepatitis; BCLC, Barcelona clinic liver cancer; cm, centimetre; G/l, grams/liter; G1, Group 1; G2, Group 2; G3, Group 3; kg, kilograms; kg/m2, kilograms/square meter; MELD, model of end-stage liver disease; mg/dl, milligrams/deciliter; ml/min/m2, milliliters/minute/square meter; mmol/l, millimole/liter; nA, not applicable; ns, nonsignificant; PBC, primary biliary cholangitis; PSC, primary sclerosing cholangitis; sec, second; U/l, Units/liter.

**TABLE 2:** Patients with tissue samples characteristics

	NAFLD (n = 18) Group 1	Compensated cirrhosis (n = 8) Group 2	HCC (n = 32) Group 3		*p*		
Age (y)	55 (±19.4)	59 (±10.5.2)	65 (±19.73)		G1/G2	G1/G3	G2/G3
	—	—	—		NS	0.05	NS
Sex (female %)	9 (50)	3 (37.5)	12 (37.5)		NS		
Weight (kg)	86.5 (±19.7)	73.4 (±20.2)	75.2 (±18.3)		G1/G2	G1/G3	G2/G3
	—	—	—		0.001	0.002	NS
Height (cm)	173.1 (±10.3)	170.8 (±12.3)	172.9 (±11.8)		NS		
BMI (kg/m2)	30.9 (±5.1)	27.2 (±6.1)	26.8 (±4.1)		G1/G2	G1/G3	G2/G3
	—	—	—		0.01	0.03	NS
AFLD	NA	4	11		NS		
NAFLD	18	2	12		G1/G2	G1/G3	G2/G3
	—	—	—		0.001	0.001	NS
Hepatitis C	NA	2	7		NS		
PBC/PSC/AIH	NA	NA	2		NA		
MELD	NA	8.4 (±6.1)	7.2 (±6.6)		NS		
BCLC staging	NA	NA	0	9	NA		
	—	—	A	13	—		
	—	—	B	10	—		
Leucocytes (g/l)	4.1 (±1.9)	3.9 (±1.8)	4.6 (±2.3)		NS		
Hemoglobin (g/l)	139.4 (±21.8)	129.2 (±14.4)	152.3 (±20.9)		NS		
Hematocrit (%)	0.41 (±0.07)	0.37 (±0.04)	0.37 (±0.06)		NS		
Platelet count (g/l)	299.7 (±59.5)	259.7 (±91.4)	289.8 (±62.2)		NS		
Urea (mg/dl)	35.1 (±19.3)	35.9 (±11.7)	34.9 (±18.8)		NS		
Creatinine (mg/dl)	0.7 (±0.4)	1.1 (±0.6)	0.7 (±0.3)		NS		
Filtration rate (ml/min/m2)	58.7 (±13.4)	56.7 (±16.7)	59.5 (±18.3)		NS		
Bilirubin (mg/dl)	0.7 (±0.4)	1.2 (±0.6)	0.8 (±0.9)		NS		
Sodium (mmol/l)	139.4 (±4.1)	141.3 (±4.8)	143.7 (±4.9)		NS		
AST (U/l)	43.7 (±23.9)	61.6 (±27.9)	57.6 (±34.3)		NS		
ALT (U/l)	41.8 (±21.8)	46.6 (±19.8)	39.6 (±29.8)		NS		
Gamma-GT (U/l)	71.9 (±39.3)	83.5 (±41.8)	75.5 (±37.9)		NS		
Alkaline Phosphatase (U/l)	86.2 (±42.8)	94.4 (±69.9)	82.5 (±59.7)		NS		
LDH (U/l)	222.5 (±71.8)	256.7 (±89.4)	166.9 (±82.7)		NS		
CRP (mg/dl)	0.9 (±0.5)	1.2 (±0.4)	1.1 (±0.6)		NS		
Quick (%)	91.4 (±18.5)	71.2 (±17.7)	79.6 (±19.4)		G1/G2	G1/G3	G2/G3
	—	—	—		0.01	0.05	NS
INR	1.1 (±0.5)	1.3 (±0.4)	1.0 (±0.9)		G1/G2	G1/G3	G2/G3
	—	—	—		0.02	0.03	NS
PT (sec)	32.9 (±4.1)	41.2 (±7.6)	38.3 (±6.3)		G1/G2	G1/G3	G2/G3
	—	—	—		0.01	0.02	NS
Fibrinogen (mg/dl)	331.0 (±63.7)	300.3 (±88.5)	312.4 (±78.9)		NS		
Albumin (mg/dl)	4270.8 (±389.8)	3570.9 (±469.9)	4080 (±369.5)		G1/G2	G1/G3	G2/G3
	—	—	—		0.03	0.02	NS
Alpha-1-Fetoprotein (µg/l)	3.2 (±0.7)	8.1 (±4.9)	25.7 (±20.8)		G1/G2	G1/G3	G2/G3
	—	—	—		NS	0.0001	0.001
Laxative use (%)	2 (11)	0 (0)	2 (6.25)		NS		
PPI use (%)	2 (11)	0 (0)	4 (12.5)		NS		
Type 1 /Type 2 diabetes (%)	6 (33)	1 (12.5)	2 (6.25)		G1/G2	G1/G3	G2/G3
	—	—	—		NS	0.01	NS

Notes: Data are expressed as case numbers (percentage) or mean ± SD.

Abbreviations: µg/l, microgram/liter; AFLD, alcohol-associated fatty liver disease; AIH, autoimmune hepatitis; BCLC, Barcelona clinic liver cancer; cm, centimeter; G/l, grams/liter; G1, Group 1; G2, Group 2; G3, Group 3; kg, kilograms; kg/m2, kilograms/square meter; MELD, model of end-stage liver disease; mg/dl, milligrams/deciliter; ml/min/m2, milliliters/minute/square meter; mmol/l, millimole/liter; nA, not applicable; ns, nonsignificant; PBC, primary biliary cholangitis; PSC, primary sclerosing cholangitis; sec, second; U/l, U/liter.

### Ethical consideration

All research was conducted in accordance with both the Declarations of Helsinki and Istanbul. The institutional ethics commission (Ethics Commission of the Medical University of Innsbruck), with an amendment to AN2017-0016 369/4.21, approved the study protocol, and written consent was given in writing by all subjects.

### Bacterial quantification by quantitative PCR

Real-time PCR amplification was performed using 16S universal primers that target the V3–V4 region of the bacterial 16S ribosomal gene. The qPCR step was performed in triplicate on a VIIA 7 PCR system (ThermoFisher, Waltham, MA, USA) with SYBR Green technology. The specificity of all qPCR products was assessed by systematic analysis of a post-PCR dissociation curve performed between 60°C and 95°C. The absolute number of copies of the 16S rRNA gene was determined by comparison with a quantitative standard curve generated by serial dilution of plasmid standards. The total 16S rRNA gene count was normalized by mg of tissue or ml of plasma.

### Statistical analysis

Data were expressed as mean ± SEM or as median with first and third quartiles. For comparing means of quantitative variables between 2 groups, the nonparametric Mann-Whitney *U* test was used. If not otherwise stated, a *p* value of <0.05 was considered statistically significant, also in cases where *p* values were false-discovery-rate-corrected. All statistical analyses were performed using *R* version 4.2.2. Please see the Supplemental data document, http://links.lww.com/HC9/A326, for details on further statistical analyses, that is, on differential gene expression and microbial abundance analysis.

### Data availability

Microbiome sequencing data are available at the European Nucleotide Archive (ENA, accession number: PRJEB54571). Transcriptome data (gene counts) and all scripts used for data analysis, statistics, and visualizations are provided through the GitHub repository https://github.com/Waschina/HCC_microbiota2022.

More detailed information for material and methods is available in the Supplemental data, http://links.lww.com/HC9/A326.

## RESULTS

### Decreased gut microbial diversity and enrichment of gut microbiota in liver tissue as features of HCC and cirrhosis

**FIGURE 1:**
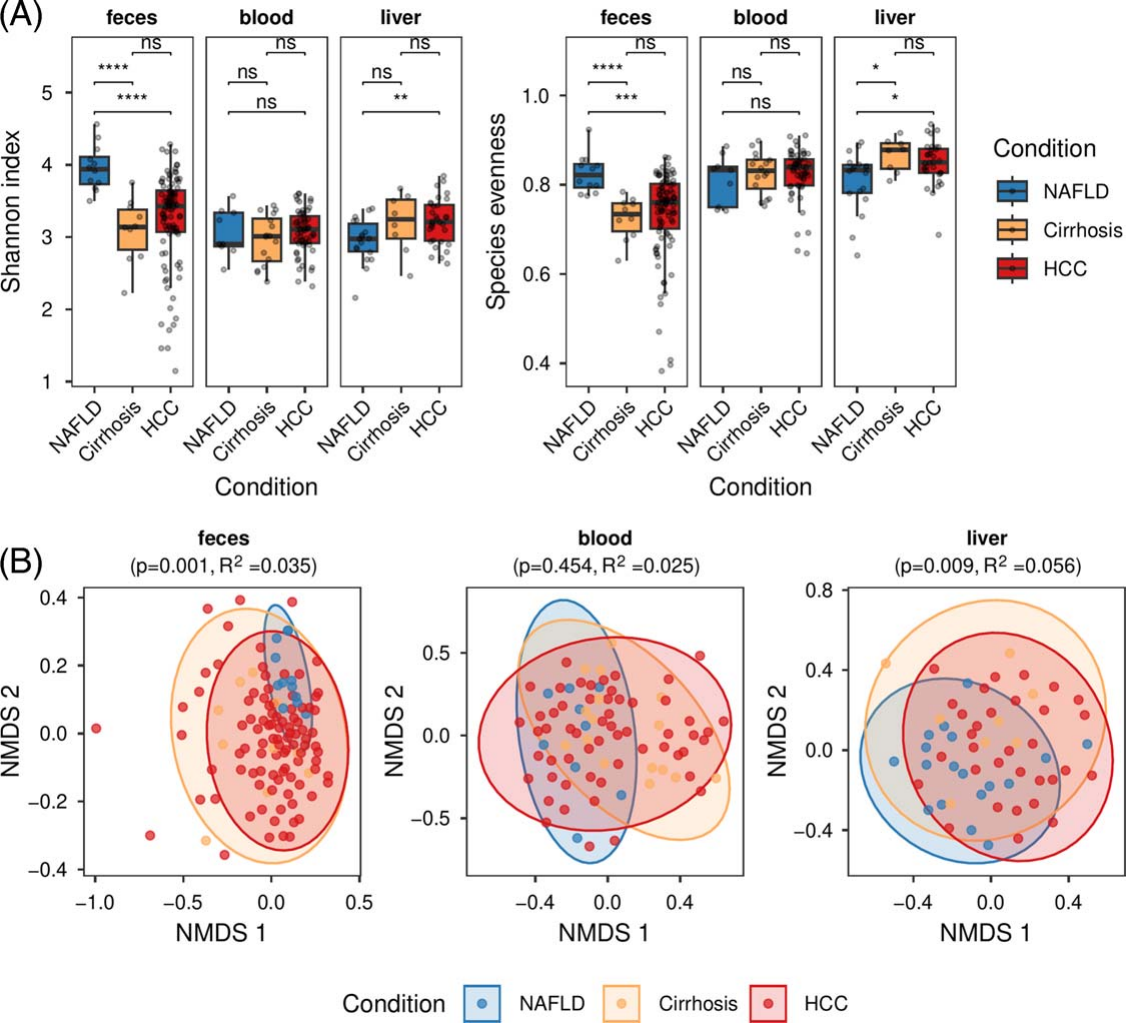
Alpha (A) and beta (B) diversities of fecal, blood, and liver microbiota. (A) Brackets are labeled with the *p* values obtained from the Mann-Whitney *U* test for pairwise group comparisons on 2 alpha diversity metrics: Shannon index and species evenness. (B) Nonmetric multidimensional scaling (NMDS) analysis of Bray-Curtis beta diversities and group separation analysis through Permutational Multivariate Analysis of Variance (PERMANOVA) using distance matrices. Global PERMANOVA *p* values and *R* ^2^ values are stated on top of each panel. As post hoc test, pairwise-PERMANOVA was performed, which revealed significant separation of NAFLD to the other 2 disease groups in feces (NAFLD-vs-HCC: *p* = 0.001, *R* ^2^ = 0.032; NAFLD-vs-cirrhosis: *p* = 0.003, *R* ^2^ = 0.1) and liver tissues (NAFLD-vs-HCC: *p* = 0.032, *R* ^2^ = 0.035; NAFLD-vs-cirrhosis: *p* = 0.008, *R* ^2^ = 0.077), but not in blood samples. Abbreviation: NMDS, nonmetric multidimensional scaling.

Intestinal dysbiosis and translocation of intestinal bacteria potentially also into the systemic bloodstream are hallmarks of CLD. We hypothesized that systematic tracing of consecutive bacterial translocation (intestine -> blood -> liver) on the microbiome level can generate insight into the role of the microbiome in the pathogenesis of HCC and, therefore, generated 16S rRNA profiles from feces, blood, and liver tissue from noncirrhotic NAFLD (n = 21), cirrhosis of various aetiologies (n = 27), and HCC (n = 111) patients (Figures S1, http://links.lww.com/HC9/A326 and S2, http://links.lww.com/HC9/A326). To understand whether gradual stages of liver disease are reflected by the level of microbial diversity, we assessed alpha diversity (Shannon, Species evenness) in feces, blood, and liver. We observed that patients with HCC and cirrhosis demonstrated reduced alpha diversity only in the intestinal microbiome, when compared to NAFLD patients (Figure 1A). In addition, we found a significant difference in liver-derived community species evenness between NAFLD and cirrhosis/HCC patients, whereas no significant difference was found at the level of blood-derived microbiota (Figure 1A). In addition to the diminished species richness, as shown by alpha diversity, we further assessed the microbial community structure and composition by measuring beta diversity (Figure 1B). We observed that fecal and liver, but not blood microbiota, significantly separated cirrhosis and HCC patients from NAFLD based on beta diversity (Figure 1B). Notably, we were unable to identify a significant discrimination between microbial signatures in patients with HCC and cirrhosis in all three tissues (ie, feces, blood, and liver). Quality control of 16S rRNA gene sequencing data included the comparison of data from biological samples with negative controls for sample preparation steps and PCR amplification (Supplemental Figure S3, http://links.lww.com/HC9/A326).

### Translocation of fecal microbiota into the bloodstream and liver in patients with cirrhosis and HCC

**FIGURE 2:**
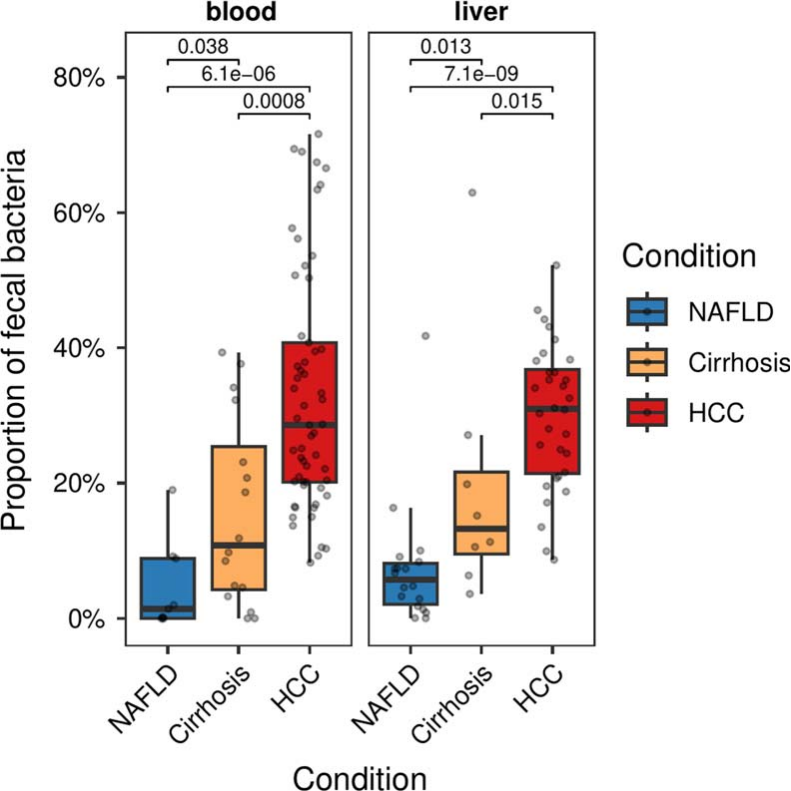
Proportion of fecal bacteria in blood and liver 16S samples. The proportion of fecal bacteria is defined as the summed relative abundance of genera, which have a relative abundance of ≥0.1% in at least 5% of the fecal samples from the same disease condition group (NAFLD, cirrhosis, and HCC). The statistical significance of pairwise group comparisons was assessed using the Mann-Whitney *U* test with NAFLD as a reference.

As patients with HCC and cirrhosis show significant alterations in the microbiome diversity of the fecal microbiota compared to NAFLD, we next tested the hypothesis that gut bacterial translocation in advanced-stage liver disease leads to the enrichment of fecal bacteria in the blood and liver tissue.^30,31^ We, therefore, assessed the degree of bacterial translocation by measuring the percentage of fecal bacteria found in the blood and liver microbiome. The proportion of fecal bacteria was defined as the summed relative abundance of 16S copies classified as genera, which have a relative abundance of ≥0.1% in at least 5% of the fecal samples from the same disease condition group (NAFLD, cirrhosis, and HCC). We observed that patients with cirrhosis or HCC presented with significantly increased numbers of fecal bacteria in blood and liver tissue compared to NAFLD patients (Figure 2). Patients with HCC displayed a higher enrichment of fecal bacteria in blood and liver samples compared to cirrhosis patients (Figure 2). We performed the same analysis on a subset of the data that included only NAFLD patients and patients with HCC or cirrhosis who had NAFLD as an underlying condition (Supplemental Figure S4, http://links.lww.com/HC9/A326), which also showed significant enrichment of fecal bacterial 16S copies in blood and liver of HCC patients compared to non-HCC NAFLD patients. Notably, we were not able to cultivate strains of bacteria in HCC tissue (n = 9) under aerobic and anaerobic conditions (data not shown). However, these findings indicated disease-specific bacterial translocation from the gut into the blood and liver.

### Identification of tissue-specific microbial signals

**FIGURE 3:**
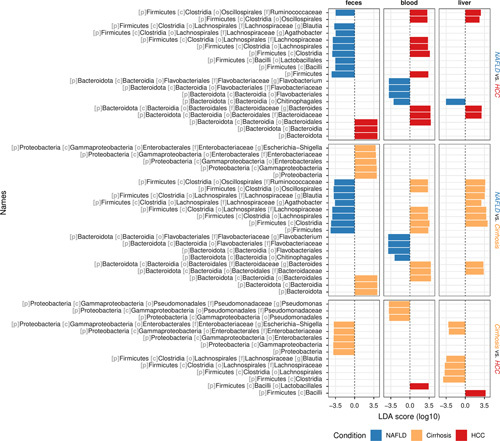
Differential analysis of the relative abundance of bacterial genera depending on the disease condition. Differential analysis of taxa relative abundance was performed using Linear discriminant analysis Effect Size (LefSe) analysis^32^ to identify taxa that most likely differentiate disease conditions in the pairwise comparisons [NAFLD versus HCC (top panel), NAFLD versus cirrhosis (middle), cirrhosis versus HCC (bottom)]. Per pairwise comparison, results are only reported for taxa, which have, in at least one of the three sample types (feces, blood, and liver), an absolute log10 LDA score of ≥3.5.

Next, we aimed at identifying bacterial taxa that are specifically enriched or depleted in a disease-specific manner and, thus, assessed the abundance of fecal, blood, and liver tissue microbiota using the linear discrimination analysis–based approach *LefSe*.^32^ In line with our previous observation, we noted that specific taxa increased in blood and liver microbiota of HCC and patients with cirrhosis compared to patients with noncirrhotic NAFLD (Figure 3). For example, the bacterial families Ruminococcaceae and Bacteroidaceae were enriched in the blood and liver of HCC and cirrhosis patients alike compared to patients with noncirrhotic NAFLD. Both families are commonly highly abundant taxa found in the human fecal microbiome. In contrast, fecal samples from cirrhosis and HCC patients both showed reduced abundance for several taxa, including known short-chain fatty acid–producing genera, such as *Blautia* and *Agathobacter* (Figure 3). Composition plots for the relative abundance of bacteria on the class level are represented in Supplemental Figure S2, http://links.lww.com/HC9/A326. Our data point toward a gross breakdown of the gut barrier and translocation of fecal microbiota in patients with HCC and cirrhosis, which results in a heterogenous enrichment of gut microbial signatures in the blood and liver.

### HCC is associated with tissues-specific change in host-microbe interaction

**FIGURE 4:**
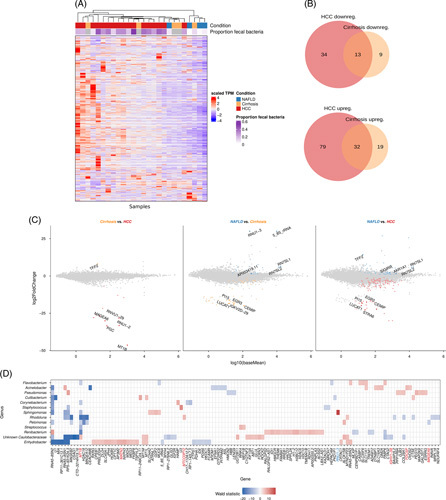
Liver biopsy transcriptome analysis. (A) Heatmap of gene expression data in transcripts per million (TPM) values unit-scaled per sample (columns). (B) Overlap of differentially expressed genes in HCC and cirrhosis with NAFLD as gene expression reference. (C) MA plots of differential gene expression analysis for all 3 pairwise contrast of the three disease conditions. The x-axis represents the log_10_ transformed average gene expression, and the y-axis represents the log_2_ fold-change due to the specific disease condition. Colored points denote genes that are overexpressed in the corresponding disease condition (color code in plot titles). The top differentially expressed genes (max. 5) in each direction are highlighted with more color saturation and the gene name as a label. (D) Statistical associations of relative abundances of bacterial genera in liver samples from HCC patients with the expression levels of specific genes. Associations are colored according to the association direction based on the Wald statistics only if the statistical associations had a significant level of p_FDR_ < 0.05. Gene names are only provided for those genes, which were differentially expressed (red: upregulated; blue: downregulated) in HCC compared to NAFLD. Only genes and bacterial genera are reported, which have, in at least 1 genus-gene combination, a significant association. The analysis was limited to genera that had, in at least 5 samples, a nonzero read count.

As patients with HCC and cirrhosis are defined by: (i) decreased alpha diversity of the fecal microbiome and (ii) increased abundance of fecal microbiota in the liver tissue, we aimed at elucidating a potential role for microbial signatures in controlling tissue-specific transcription. To do so, we performed bulk RNA sequencing of liver specimens (NAFLD, n = 4; liver cirrhosis, n = 5; and HCC, n = 17). Using principal component analysis, we observed a separation between the transcriptome of HCC and NAFLD patients (Supplemental Figure S5, http://links.lww.com/HC9/A326). By assessing globally differentially expressed genes, we observed distinct expression patterns between NAFLD versus cirrhosis and HCC, whereas cirrhosis and HCC did not separate distinctly. As we observed an increased abundance of fecal bacteria in liver tissue of HCC/cirrhosis (Figure 2), we also clustered host transcriptome data in accordance with the proportion of intestinal microbial communities found in the same patient sample through 16S rRNA amplicon sequencing. Again, we were not able to identify a global influence of fecal bacteria on tissue gene expression (Figure 4A). We further aimed to identify disease-specific gene expression patterns and observed only very minor changes between HCC and cirrhosis (Figure 4B, C). In addition, Gene Ontology term enrichment analysis for: (i) biological processes, (ii) cellular components, or (iii) molecular functions revealed a strong, gradual overlap of transcriptional signatures that distinguish HCC and cirrhosis from NAFLD tissue (Supplemental Figure S6, http://links.lww.com/HC9/A326). Having shown that neither the microbial nor the transcriptional expression signature alone is informative to sufficiently discriminate HCC from cirrhosis tissue, we wondered whether the host-microbial crosstalk is specifically altered in HCC liver tissue. We, therefore, aimed to identify those microbial genera in liver samples, whose relative abundance is associated with the gene expression in the liver tissue of HCC patients. To do so, we performed negative binomial generalized linear model fitting and Wald statistics to identify genes, whose expression levels could be explained by the relative abundance of specific genera. By doing so, we identified numerous microbial genera that directly or indirectly correlated with the expression of genes, which were also identified as significantly upregulated in HCC compared to NAFLD (Figure 4D). Interestingly, we identified a strong association of microbial abundance with the MT1B gene, which encodes metallothionein.^33^ In our cohort, MT1B is overexpressed in HCC compared to cirrhosis and NAFLD. Metallothionein plays an important role as scavenger of reactive oxygen species and is upregulated under oxidative stress.^34^ The metallothionein expression has been described in HCC and diethylnitrosamine-induced liver tumors in mice.^35^ Notably, the association of microbial abundance with host gene expression was not specific to a distinct genus or ASV (data not shown). Hence, our data point toward a potential site-directed interaction between the fecal microbiota in the liver that might influence host gene expression and, thereby, fuel the transition from cirrhosis into the manifestation of HCC.

## DISCUSSION

The gut microbiota and its metabolites have been proposed as cofactors in liver disease progression and the development of HCC through their interaction with immune compartments through the gut-liver axis.^21^ Today, a systematic analysis of disrupted host-microbial interaction along the gut-liver axis is lacking for patients with HCC. In this study, we thoroughly tracked fecal, blood, and liver tissue 16S rRNA signatures from patients with HCC, cirrhosis, and NAFLD to identify alterations of the microbiome that would discriminate between these conditions. In addition, we assessed the liver transcriptome of patients with HCC, cirrhosis, and NAFLD patients to understand whether specific tissue-specific transcripts may link to bacterial signatures, which would allow us to identify the perturbation of host-microbe interactions in advanced liver disease.

By doing so, we found a distinct difference in the gut microbiota between NAFLD and HCC/cirrhosis, which is in line with previous reports showing a distinct clustering of the gut microbiota between patients with cirrhosis with HCC or patients with cirrhosis without HCC.^28,36^ More importantly, we identified significantly different alpha diversity levels in the liver tissue between NAFLD and cirrhosis/HCC patients, indicating that the tissue-specific microbiome is indeed changing in a disease stage-dependent manner. It has been postulated in previous studies that liver disease in humans might associate with changes in the microbiome in the liver and circulation.^37,38^ In this context, Schierwagen and coworkers found evidence for a circulating microbiome in the buffy coat fraction of peripheral and portal and central venous blood from 7 patients with liver cirrhosis undergoing transjugular portosystemic shunting.^39^ They detected *Proteobacteria* sequences in the blood, and in some patients, they could cultivate the bacterium, suggesting that circulating DNA sequences were derived from living organisms. For NAFLD-associated liver fibrosis, a specific blood microbiome signature was described in a small cohort of patients with severe obesity.^40^ Sookoian and coworkers studied the liver tissue 16S *rRNA gene* bacterial metataxonomic signature in 2 cohorts of NAFLD patients.^30^ This was also confirmed in other NAFLD studies.^41^ In accordance, Leinwand and coworkers showed that Proteobacteria are enriched in the liver microbiome, and microbial changes are associated with substantial changes in hepatic immune cells.^42^ In contrast to these studies, we here present a comprehensive analysis of the gut-blood-liver axis by assessing the microbiome within the gut, blood, and liver. Importantly, we analyzed HCC and not adjacent tissue, as the microbial signature may differ in these 2 entities as already shown in pancreatic cancer.^43^ Our data indicate that DNA from fecal bacteria is enriched in the posthepatic blood and liver tissue from patients with HCC and cirrhosis with an increase in more advanced liver disease, as significantly enriched in comparison to NAFLD patients. Importantly, none of the patients suffered from decompensated cirrhosis, including acute on chronic liver injury, as these facts may change the intestinal microbial disturbances.^44^ These data might point to a common pathophysiological sequence of increased gut permeability in both cirrhosis and HCC patients, which could fuel disease progression. In addition, these data also indicate that there is not a single liver-specific bacterial strain that is enriched in HCC patients compared to cirrhosis that would explain a direct causal contribution of a single bacterium toward the malignant transformation into HCC. Notably, we were unable to cultivate single bacterial strains from HCC tissue under aerobic or anaerobic conditions. This observation suggests that the identified bacterial 16S rRNA gene sequences are not representing live bacteria but rather bacterial fragments (DNA) that are translocated into the liver.

To understand the underlying consequences of an increased abundance of fecal microbiota in the liver of cirrhosis/HCC patients, we aimed to integrate the microbial signature into the disease-specific transcriptional signatures of the host. Our findings indicate that the transcriptional profile is distinct between NAFLD and cirrhosis or HCC patients. These findings are in line with different studies showing specific genes, pathways, and functional terms differentially regulated in HCC compared to NAFLD or healthy patients.^45–47^ A very recent study^48^ could detect a specific microbial tissue or transcriptome signature in HCC of HBV patients compared to a healthy control group. Within our study, we further examined whether there is a specific HCC signature, which can be uniquely distinguished from cirrhotic patients. An experimental study indicates a potentially immediate effect of enteric bacterial translocation on liver antitumor immunity.^49^ Interestingly, we were not able to disentangle either a microbial or a transcriptional signature that uniquely attributes to HCC patients. Nevertheless, we were able to identify significant associations between the abundance of enteric bacteria within the liver and the expression of host genes that were upregulated in HCC tissue. Among others, we identified a significant association of several bacterial genera with metallothionein MT1B, which has been previously shown to be strongly upregulated in HCC patients.^50^ Altogether, our data identify a liver-specific disturbance of host-microbiota interaction in HCC patients, which seems to arise from an increased perturbation of the gut barrier in these patients.^21^ The gradual increase in host-microbe perturbation in patients with cirrhosis and HCC (compared to NAFLD) warrants further research to understand the underlying molecular mechanisms and consequences of enteric microbes invading liver tissue.

## Supplementary Material
