# Supplementary material for: A gut bacterial signature in blood and liver tissue characterizes cirrhosis and hepatocellular carcinoma
Source: Hepatol Commun. 2023 Jun 14;7(7):e00182. doi: 10.1097/HC9.0000000000000182 (PMC10270494; doi:10.1097/HC9.0000000000000182)
Supplement: Supplementary file 1 [file hc9-7-e00182-s001.docx]

**A gut bacterial signature in blood and liver tissue characterizes cirrhosis and hepatocellular carcinoma**

**Supplementary Data**

Maria Effenberger MD^1+^, Silvio Waschina PhD^2+^, Christina Bronowski MSc PhD^3^, Gregor Sturm MSc^4^, Oronzo Tassiello^2^, Felix Sommer PhD^3^, Andreas Zollner MD^1^, Christina Watschinger MSc^5^, Felix Grabherr PhD^1^, Ronald Gstir PhD^6^, Christoph Grander PhD^1^, Barbara Enrich^1^, Reto Bale MD^7^, Daniel Putzer MD^7^, Angela Djanani MD^1^, Alexander R Moschen PhD^5,8^, Heinz Zoller MD^1^, Jan Rupp PhD^9^, Stefan Schreiber PhD^3,10^, Remy Burcelin MD^11^, Cornelia Lass Flörl MD^6^, Zlatko Trajanoski PhD^4^, Georg Oberhuber MD^12^, Philp Rosenstiel PhD^3^, Timon E Adolph PhD^1^, Konrad Aden MD^3,10*^and Herbert Tilg MD^1*^

^+^ Contributed equally to this work

^*^ These authors share senior authorship

1. Department of Internal Medicine I, Gastroenterology, Hepatology, Endocrinology & Metabolism, Medical University of Innsbruck, Innsbruck, Austria

2. Institute for Human Nutrition and Food Science, Division of Nutriinformatics, Christian-Albrechts-University of Kiel, Kiel, Germany

3. Institute of Clinical Molecular Biology, Christian-Albrechts-University and University Hospital Schleswig-Holstein, Campus Kiel, 24105 Kiel, Germany

4. Biocenter, Institute of Bioinformatics, Medical University of Innsbruck, Innsbruck, Austria

5. Department of Internal Medicine I, Gastroenterology, Nephrology, Metabolism & Endocrinology, Johannes Kepler University, Linz, Austria

6. Institute of Hygiene and Medical Microbiology, ECMM, Medical University of Innsbruck, Innsbruck, Austria

7. Department of Radiology, Medical University of Innsbruck, Innsbruck, Austria

8. Christian Doppler Laboratory for Mucosal Immunology, Johannes Kepler University, Linz, Austria

9. Department of Infectious Diseases and Microbiology, University Hospital Schleswig-Holstein, Luebeck, Germany

10. Department of Internal Medicine I, Christian-Albrechts-University and University Hospital Schleswig-Holstein, Campus Kiel, 24105 Kiel, Germany

11. INSERM 1297 and University Paul Sabatier: Institut des Maladies Métaboliques et Cardiovasculaires, INSERM U1048 F-31432 Toulouse, France and Université Paul Sabatier, F-31432, Toulouse, France

12. INNPATH, Institute of Pathology, University Hospital of Innsbruck, 6020 Innsbruck, Austria

*Corresponding author:*

Herbert Tilg, M.D., Department of Internal Medicine I, Gastroenterology, Hepatology, Endocrinology & Metabolism, Medical University Innsbruck, Innsbruck, Austria

Phone: +43 512 504 23539, Fax: +43 512 504 23538, E-mail: herbert.tilg@i-med.ac.at

and

Konrad Aden, M.D. Department of Internal Medicine I., University Medical Center Schleswig-Holstein, Campus Kiel; Rosalind-Franklin-Strasse 12, 24105 Kiel, Germany, phone: +49 (431) 500-22455, fax: +49 (431) 500-15104, [k.aden@ikmb.uni-kiel.de](mailto:k.aden@ikmb.uni-kiel.de)

**Materials and Methods**

*Patients*

Hepatocellular carcinoma (HCC) patient group

Tumors with a score of Liver Imaging Reporting and Data System (LI-RADS®) 5 were defined as HCC^1-3^ and were additionally clinically staged using the Barcelona Clinic Liver Cancer Staging (BCLC) System. All patients included were treatment naïve.

The numbers of HCC patients eligible for stereotactic radiofrequency ablation (RFA) and tumor tissue sampling in cirrhosis (n=32) were as follows: 9 patients were BCLC stage 0, 13 patients stage A and 10 patients fitted the criteria for BCLC stage B. The underlying disease in the HCC group eligible for RFA was alcoholic fatty liver disease (AFLD) in 11 patients, non-alcoholic fatty liver disease (NAFLD) in 12 patients and 7 had hepatitis C (HCV) infection. Furthermore, one patient with primary biliary cholangitis and another patient with autoimmune hepatitis were included in the HCC group.

In 79 patients HCC patients we were only able to collect fecal and blood samples. 12 patients were BCLC stage 0, 28 patients stage A and 25 patients fitted the criteria for BCLC stage B and 14 patients were stage C. The underlying disease in the HCC group not eligible for RFA was AFLD in 34 patients, NAFLD in 19 patients and 13 had hepatitis B (HBV) and 6 patients with HCV infection were included. Furthermore, 5 patients with primary biliary cholangitis and 2 patients with autoimmune hepatitis were included in the HCC group.

Liver cirrhosis patient group (different etiologies)

Liver cirrhosis (n=27) was confirmed by abdominal computed tomography (CT) and indirect clinical signs of cirrhosis signs, such as esophageal varices, portal hypertension, ascites, hepatic encephalopathy or thrombocytopenia. The model of end stage liver disease (MELD) score and the Child Pugh (CP) score were calculated. All patients included in the study suffered from compensated cirrhosis. Liver biopsy was performed in 4 patients with AFLD, 2 patients with NAFLD and 2 patients with HCV infection.

NAFLD patient group

NAFLD (n= 21) was defined by imaging evidence of hepatic steatosis and lack of secondary causes of hepatic fat accumulation, like long-term use of steatogenic medication or significant alcohol consumption^4,5^. Furthermore, FIB-4 score was performed in patients with suspected NAFLD^6^. Biopsy was performed in 18 of 21 patients according to the NAFLD activity score (NAS)^7^ (Six with NAS 3, four with NAS 2, seven 7 with NAS 4 and one with NAS of 5). None of these patients had cirrhosis. Liver biopsies were examined and diagnosed by two independent pathologists. The patient’s selection is recaptured in Supplementary Figure 1a.

In 16 HCC-; 3 cirrhosis and 8 NAFLD patients we collected blood, feces and liver tissue samples. Type 1 or type 2 diabetes was defined by using antidiabetic drugs. Exclusion criteria for study inclusion were acute liver injury/failure, acute on chronic liver failure, liver transplantation, multiorgan failure, uncontrolled infection, acute decompensation within the last 12 months, active upper gastrointestinal bleeding, chronic renal failure Grade III, pregnancy, treatment with antibiotics within 3 months before study inclusion, active alcohol use within the last 6 months and ongoing uncontrolled drug abuse. Decompensated cirrhosis was defined by ascites, bleeding, encephalopathy, and jaundice^8^. In the NAFLD group, patients with other underlying liver diseases (hepatitis B or C, autoimmune liver disease, primary biliary cholangitis and primary sclerosing cholangitis, Wilson disease, hemochromatosis and suspected drug-induced liver injury) or HIV-infection were excluded.

*Clinical findings of hepatic steatosis, cirrhosis and HCC*

Hepatic steatosis and ascites were diagnosed by abdominal ultrasound examination. Two experienced physicians (each > 3000 US-exams) used the Philips EPIQ 5^®^ ultrasound machine (Philips Corporation, Amsterdam, The Netherlands). Hepatic encephalopathy was diagnosed by using the West Haven criteria in combination with the Psychometric Hepatic Encephalopathy score as described elsewhere^9^.

*Blood samples*

Peripheral whole, post-hepatic blood (n=83) (HCC n=58, cirrhosis n=16, NAFLD n=9) samples were taken from patients during the periodical medical monitoring. Serum was prepared within 2 hours after the blood draw by centrifugation at 1200 g for 15 minutes at 20°C in a universal 320/320R centrifuge (Hettich, Tuttlingen, Germany). Serum was stored at -80°C.

*Fecal samples*

Patients (n=118) (HCC n=96, cirrhosis n=10, NAFLD n=12) collected 50 mg of their stool and put into 3 sterile tubes, which where send to them in advance. They immediately transferred the tubes to −20 °C storage (fresh frozen) and the cold chain storage maintained at −20 °C using transportable cool bags until reaching the laboratory, where tube samples were transferred to −80 °C. Participants completed a Bristol Stool Scale questionnaire during their periodical medical monitoring.

*Liver biopsies via RFA*

RFA was performed as described^10^ during general anesthesia, muscle relaxation, and single shot broadband antibiotics. A contrast‐enhanced (100‐150 mL of Iopromide (Ultravist 370; Schering AG, Berlin, Germany), 370 mg I/mL, 3 mL/s) helical CT (Somatom Open; Siemens, Erlangen, Germany) with a 3 mm slice thickness was performed. Images are obtained 35‐40 and 70‐80 seconds after initiation of contrast media injection, representing the late arterial and late portal phases.

Multiple electrode probe positions were planned on the 3D‐CT data set with the software of the Treon optical frameless stereotactic navigation system (Medtronic Inc., Louisville, KY). The goal was to cover the entire tumor volume with overlapping necrosis. The Atlas aiming device (Elekta Inc., Schwabmünchen, Germany) was manually adjusted using the guidance software of the navigation system after skin fiducial‐based registration, an accuracy check, and scrubbing and draping. Coaxial needles (15‐G, Bard Inc., Covington, GA) were sequentially advanced through the targeting device to the preplanned depth during repeated temporary breath holds. To verify correct needle positions, one unenhanced CT with all coaxial needles in place was taken.

Thereafter, liver biopsy samples were taken. Three RFA probes with a 3‐cm active tip were inserted through the shorter coaxial needles, the latter being retracted to uncover the active probe exposure. At each position, ablations were performed using the switching control mode for up to three probes during the 16‐minute ablation per cycle.

After hot withdrawal of all probes, a contrast‐enhanced control CT in the late arterial and portal phase was obtained to exclude treatment‐related complications (e.g., bleeding, pneumothorax) and confirm sufficient coverage of the tumor by the ablation zone. Within 24 hours of treatment, a US examination was performed to exclude delayed treatment‐related complications^11^.

All the samples were stored in a sterile container and kept at -80°C until assayed.

*Ultrasound-guided percutaneous liver biopsy (UPLB)*

Antiplatelet drugs or oral anticoagulation therapy was set on hold one week prior to UPLB. In 8 patients with cirrhosis and 18 patients with NAFLD this procedure was performed. At this institution, no pre-procedure sedation is used. One experienced physician (> 3000 US-exams and >100 UPLBs) did the biopsies using the Philips EPIQ 5® ultrasound machine (Philips Corporation, Amsterdam, The Netherlands). The site for UPLB was identified by US in all cases, usually at an intercostal space in the anterior axillary line. Under US guidance, 10 ml of 1% lidocaine were injected percutaneously and with adequate infiltration of the liver capsule. UPLB was performed using an 18 G Temno II semi-automatic tru-cut biopsy needle (Cardinal Health, Dublin, OH). All the samples were stored in a sterile container and kept at -80°C until assayed. Patients were monitored after the procedure for 6h. After a final blood- and US-check they were discharged. All patients were follow-up in 2 weeks to review the results of histology.

*Biochemical analyses*

For all human biochemical analyses, we used the Cobas® 8000 analyzer (Roche, Basel, Switzerland) according to the manufacturer´s specification.

*Histology*

HE-staining of liver sections was performed by INNPATH (Institute of Pathology, University Hospital of Innsbruck, Innsbruck, Austria) and pathologists analyzed the stained liver sections in a blinded manner concerning hepatic steatosis, inflammation, and fibrosis. Hepatic steatosis was quantified by percentage of cells showing lipid accumulation. Whenever viability of HCC was not obvious in HE sections, it was confirmed with a TUNEL assay (ApopTag; EMD Millipore Corporation, Burlington, MA). However, also areas that were considered viable in HE were retrospectively examined by a TUNEL assay.

*DNA extraction*

DNA was extracted from plasma (200 µl), liver (28–78 mg; depending on the sample type), and feces (500 µl) using an optimized blood and tissue-specific technique that was carefully designed to minimize the risk of cross-contamination. DNA was extracted using a silica-based column after three rounds of mechanical lysis for 30 s at 30 Hz in a bead beater (TissueLyser, Qiagen, Hilden, Germany) with 0.1 mm glass beads (MoBio, Qiagen, Hilden, Germany) to increase the yield of bacterial DNA. Total genomic DNA was collected in 50 µl of molecular grade water. The quality and quantity of extracted DNA were monitored by gel electrophoresis (1% w/w agarose in 0.5 x TBE buffer) and the NanoDrop 2000 UV spectrophotometer (ThermoFisher, Waltham, MA). Extracted DNA was stored at −20 °C until further processing.

*16S rRNA gene sequencing*

The V3–V4 hypervariable regions of the 16S rRNA gene (467 bp on the *E. coli* reference genome) were amplified from the DNA extracts during the first PCR step using universal primer Vaiomer 1F (CTTTCCCTACACGACGCTCTTCCGATCT–TCCTACGGGAGGCAGCAGT, partial P5 adapter–primer) and universal primer Vaiomer 1R (GGAGTTCAGACGTGTGCTCTTCCGATCT–GGACTACCAGGGTATCTAATCCTGTT, partial P7 adapter–primer), which are fusion primers based on the qPCR primers. The first PCR reaction was carried out on a Veriti Thermal Cycler (ThermoFisher, Waltham, MA) as follows: an initial denaturation step (94 °C for 10 min), 35 cycles of amplification (94 °C for 1 min, 68 °C for 1 min and 72 °C for 1 min) and a final elongation step at 72 °C for 10 min. Amplicons were then purified using the magnetic beads Agencourt AMPure XP for PCR Purification (Beckman Coulter, Brea, CA).

Sample multiplexing was performed using tailor-made 6-bp unique index sequences. These were added during the second PCR step at the same time as the second part of the P5 or P7 adapters used for the sequencing step on the MiSeq flow cells with the forward primer Vaiomer 2F (AATGATACGGCGACCACCGAGATCTACACT–CTTTCCCTACACGAC, partial P5 adapter–primer targeting primer 1F) and reverse primer Vaiomer 2R (CAAGCAGAAGACGGCATACGAGAT–index–GTGACT–GGAGTTCAGACGTGT, partial P7 adapter including index–primer targeting primer 1R). This second PCR step was performed on 50–200 ng of purified amplicons from the first PCR. The PCR reaction was carried out on a Veriti Thermal Cycler (ThermoFisher, Waltham, MA) and was run as follows: an initial denaturation step (94 °C for 10 min), 12 cycles of amplification (94 °C for 1 min, 65 °C for 1 min and 72 °C for 1 min) and a final elongation step at 72 °C for 10 min. Amplicons were purified as described for the first PCR round. All libraries were pooled at the same quantity to generate an equivalent number of raw reads within each library. Sequencing fragments were detected using MiSeq Illumina technology with 2 x 300 paired-end MiSeq kit v3.

### *Assessment of potential sample contamination*

Samples with low bacterial biomass, such as tissues and plasma, are highly susceptible to potential contamination from the environment and reagents. This would therefore give false-positive results^12,13^. The technique to account for this challenge, was published before^14^. We included a comprehensive set of negative controls to test for environmental sample contamination at major steps in the analysis. During tissue collection, tubes were kept open next to the operation field throughout the entire procedure. Contamination that derived from tissue manipulation was monitored by an additional set of tubes kept open next to the operator throughout blood centrifugation and plasma collection, as well as during tissue aliquoting. The cutting board that was used to aliquot tissue samples was sampled prior to tissue manipulation. Water samples were used to control for labware, reagent and/or environmental contamination during DNA extraction and during amplification steps for tissue 16S rRNA quantification. Beta diversity analysis of all blood samples, liver tissue samples and negative controls showed a clear separation of negative controls from blood and liver samples (Figure S4) indicating the 16S signatures obtained in this study are not due to technical bias in sample preparation or the sequencing procedure. Furthermore, we found significantly more 16S reads in liver and blood samples than in negative controls (Figure S4). After thorough validation of negative controls on a case-by-case basis, 16S rRNA quantification and sequencing data were used for the discovery of tissue-specific bacterial signatures^14^.

*16S rRNA gene sequencing data analysis*

Sequencing reads in FASTQ format were analyzed using the R-package DADA2 (version 1.20.0) according to published workflows^15^. In detail, the sequences were filtered and trimmed using the function ‘*filterAndTrim’* in its default parametrization except for the option ‘*maxEE’* which was set to 2 to discard all sequences with an expected error number of >2 bases after sequence trimming. Amplicon sequence variants (ASVs) were inferred using dada2’s core function ‘*dada*’ and bimera ASV sequences were detected and removed using the function ‘*removeBimeraDenovo*’. The taxonomic classification of ASVs were predicted using the function ‘assignTaxonomy’ using the reference training data set from SILVA (NR99, version 138.1)^16^. Alpha-diversity metrics were calculated from the ASV count table using the R-package vegan (version 2.5.7)^17^. Beta-diversities were calculated using the Bray-Curtis dissimilarity index^18^, also using the R-package vegan^17^.

Non-metric multidimensional scaling (NMDS) was applied to the calculated beta diversity values using the function ‘metaMDS’ from the *vegan*-R-package (results shown in Fig. 1B). Furthermore, permutational multivariate analysis of Variance (PERMANOVA) was performed with 999 permutations on the basis of the calculated beta diversities using the ‘*adonis’* function using all three disease entities (NAFLD, Cirrhosis, HCC) as factors. As post-hoc tests, we performed pairwise-PERMANOVA as implemented in the R-package “pairwiseAdonis”^19^(version 0.4). Differential analysis of relative taxa abundances (on the levels of phylum, class, order, family, and genus) was performed using *lefser* ^20^(version 1.6.0) and the taxonomic group relative abundance table that contained the summed ASV proportions based on the ASV taxonomy predictions from DADA2. *lefser* is an *R* implementation of the LEfSe (Linear discriminant analysis Effect Size) algorithm^21^, that is based on linear discriminant analysis and aims to identify features that discriminate two groups of samples. The function “lefser(…)” was used in its default parametrization.

*Liver biopsy transcriptome sequencing*

RNA extraction (HCC n=17, cirrhosis n=5, NAFLD n=4) was performed using RNeasy Mini Kit (Qiagen, Hilden, Germany) according to manufacturer´s instructions. Samples with an RNA Integrity Number (RIN) of 7 or higher were processed to generate libraries for mRNA sequencing following the Illumina® TruSeq Stranded mRNA Sample Preparation Guide. In this method, poly-A mRNAs were purified from 0.5 μg total RNA, fragmented and reverse-transcribed into cDNAs. Double strand cDNAs were adenylated at the 3′ ends and ligated to indexed sequencing adaptors, followed with amplification for 15 cycles. One femtomole of the sequencing libraries (median size ~260 nt) were denatured and loaded onto a flow cell for cluster generation using the Illumina cBot. Paired-end sequencing was carried out on a HiSeq 2500 sequencer (Illumina, San Diego, CA, USA)

*Transcriptome data analysis*

Transcriptome sequencing reads were preprocessed and mapped to the human genome GRCh38/hg38 ^22^ and GENCODE v38 annotations using the nf-core RNAseq pipeline version 3.9 ^23^ with the "star_salmon" quantification rout. In brief, reads were mapped using STAR version 2.7.10a ^24^ and gene expression quantified with Salmon version 1.5.2^25^. As part of this workflow, reads were trimmed using TrimGalore v0.6.7 (https://www.bioinformatics.babraham.ac.uk/projects/trim_galore/), a wrapper around cutadapt v3.4^26^. Trimmed reads were aligned to the reference genome using STAR version 2.7.10a^24^.Raw counts and transcripts per million (TPM) were quantified with Salmon v1.5.2^25^ using the aligned BAM files as input. Quality control was performed with DupRadar v1.18.0c^27^ FastQC v0.11.9, RSeQC v3.0.1^28^, QualiMap v2.2.2-dev ^29^, and Preseq v3.1.1^30^. Quality metrics were aggregated into a MultiQC report^31^. The required software dependencies were obtained as Singularity containers^32^ from the Biocontainers registry^33^.

The heatmap in Fig 4A that displays clustered transcript per million (TPM) values was generated using the R-package ‘ComplexHeatmap’ version 2.12.1 ^34^. Venn-diagrams in Figure 4B were created using the package ‘VennDiagrams’ (version 1.7.3). Differential gene expression analysis between two disease condition groups were performed using DESeq2 on the basis of gene counts. DESeq2 was run with the parameter “design = ~ Condition + Age + Sex” to test for the potential effect of the disease condition (NAFLD, Cirrhosis, HCC) on gene expression while accounting for age and patient self-reported sex as potential covariates. Pairwise differential gene expression analysis between two disease condition groups was performed with DESeq2’s default settings that includes also false discovery rate (FDR)-based p-value adjustment for multiple testing. Moreover, in order to test for the potential impact of bacteria residing in the liver tissue on host gene expression (results shown in Fig. 4D) DESeq2 was re-applied with the parameter “design = ~ ‘relative. ASV abundance’ + Age + Sex”. This test was limited to genera that had in at least 8 samples a relative abundance equal or higher than 0.1%.

Gene ontology analysis was performed using the R-package *topGO* (version 2.48.0)^35^. Sets of differentially expressed genes with an adjusted p-values below 0.05 were separated into negative and positive associations based on the DESeq2 Wald-statistic estimate sign. GO-term enrichment sets were performed using the topGO function “runTest” and the arguments *algorithm = “classic”* and *statistic = “fisher”*.

# *Statistical Analysis*

Data are presented as boxplots showing mean and the 25% and 75% quantile. Individual data points are shown if applicable. Statistical significance was assessed using the Mann-Whitney U-test (for α-diversity and proportion of fecal bacteria) and PERMANOVA (for β-diversity).

**Supplementary Figures**

**Supplementary Figure S1**
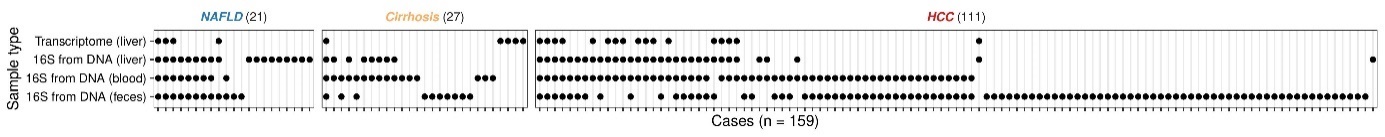


**Cohort Overview.** The study cohort consists of 159 clinical cases (21 NAFLD, 27 cirrhosis, 111 HCC). Points indicate the obtained samples for each case.

**Supplementary Figure S2**

**
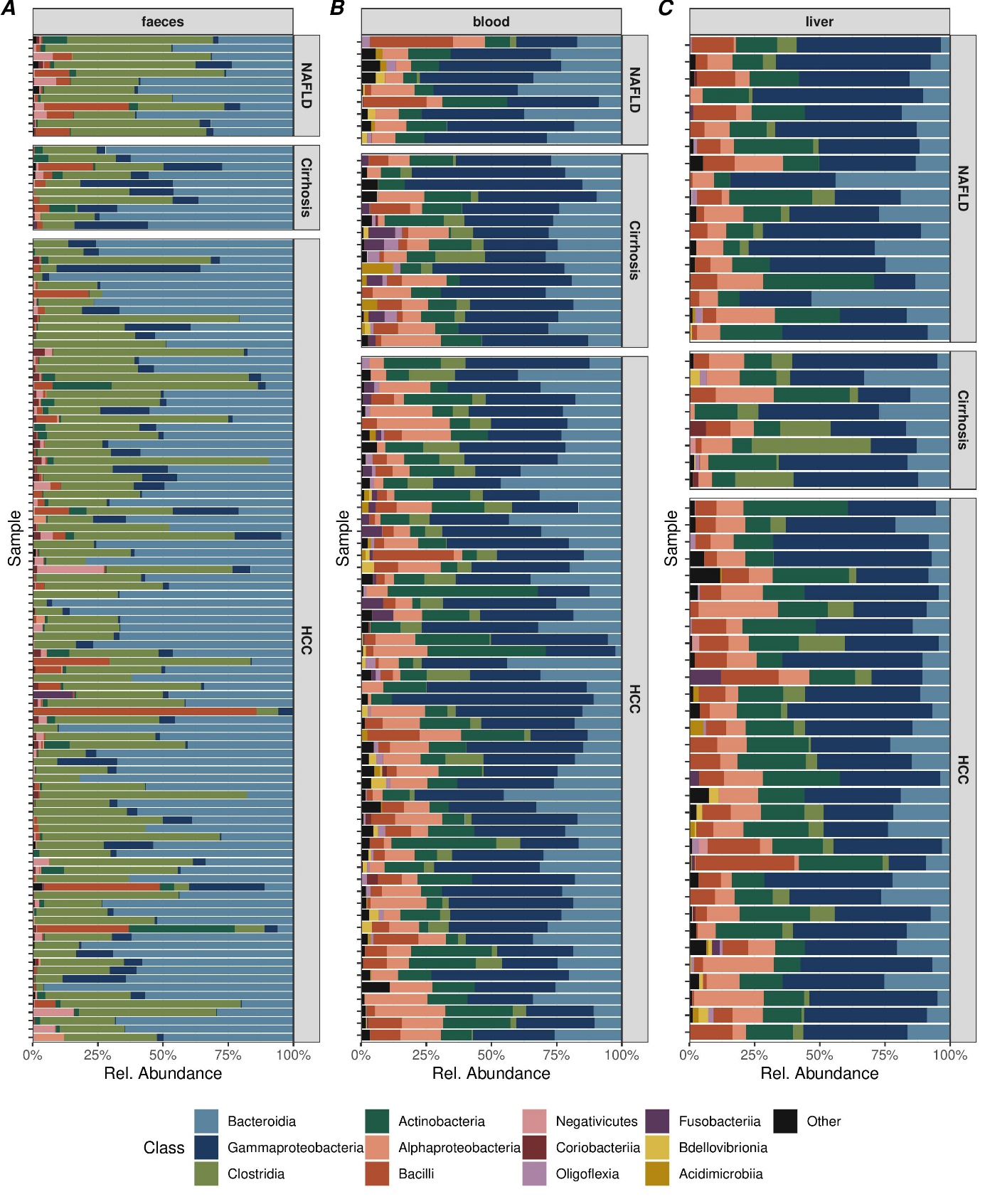
**

Composition plots for the relative abundance of bacteria on class level. Each stacked bar plot represents the composition in individual feces (A), blood (B), or (C) liver tissue samples. Samples are vertically groups by disease condition (NAFLD, cirrhosis, HCC).

**Supplementary Figure S3**


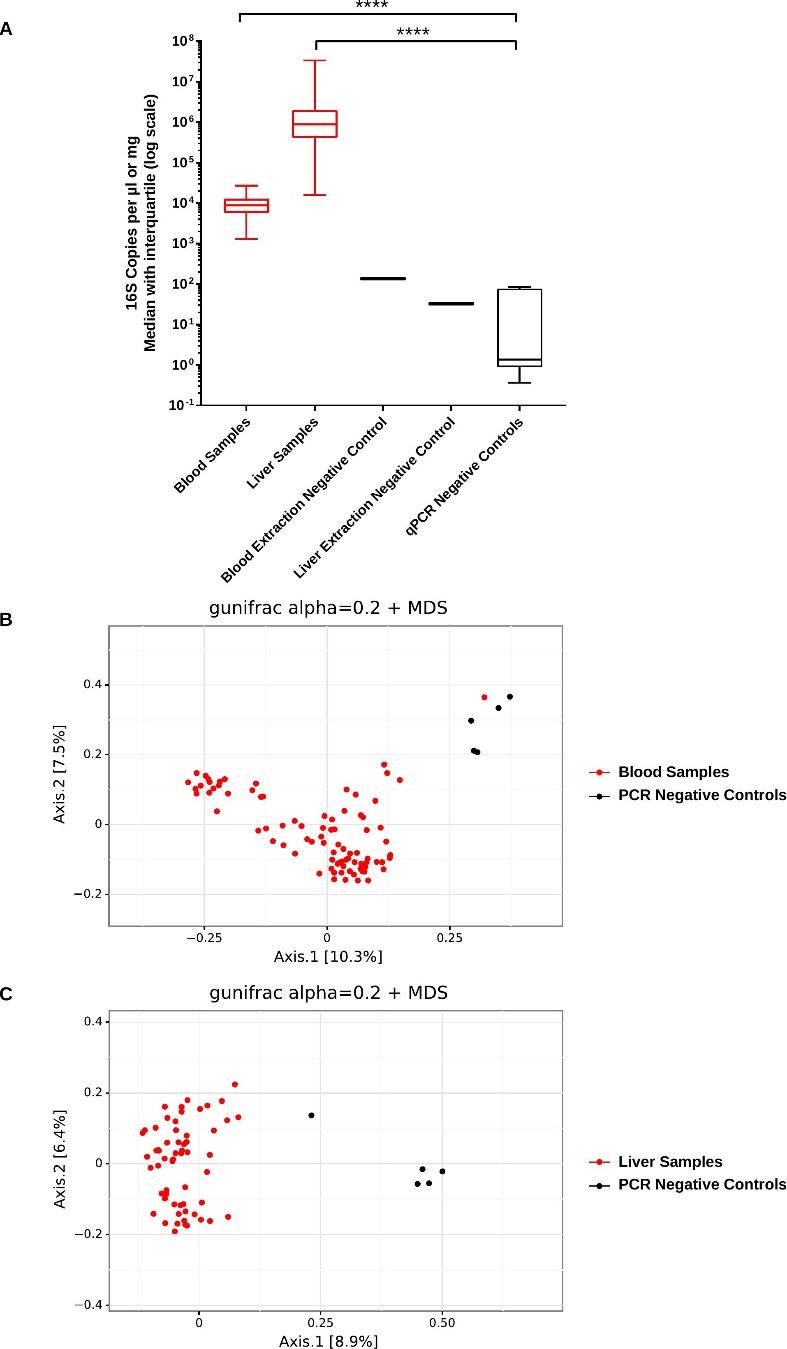


**Quality control of blood and liver 16S rRNA gene sequencing data. (A)** Comparison of the quantity of bacterial DNA (log scale) assessed by qPCR in blood samples, liver samples and negative controls. Extraction Negative control: molecular grade water added in an empty tube at the DNA extraction step and extracted and amplified at the same time as the biological samples. qPCR Negative controls: molecular grade water added in an empty tube during qPCR steps and amplified at the same time as the biological samples DNA. **** : p < 0.0001 with Kruskal-Wallis + Dunn’s post-test. **(B)** Comparison of beta diversities by ordination analysis of the 16S rRNA gene sequencing data using gunifrac dissimilarity distances of the blood samples and the PCR negative controls. The results show that one blood sample clustered with the negative controls. This specific sample has been removed from all further data analysis. **(C)** Comparison of beta diversities by ordination analysis of the 16S rRNA gene sequencing data using gunifrac dissimilarity distances of the liver samples and PCR the negative controls. PCR negative controls: molecular grade water added in an empty tube during PCR steps, amplified and sequenced at the same time as the biological samples.

**Supplementary Figure S4**


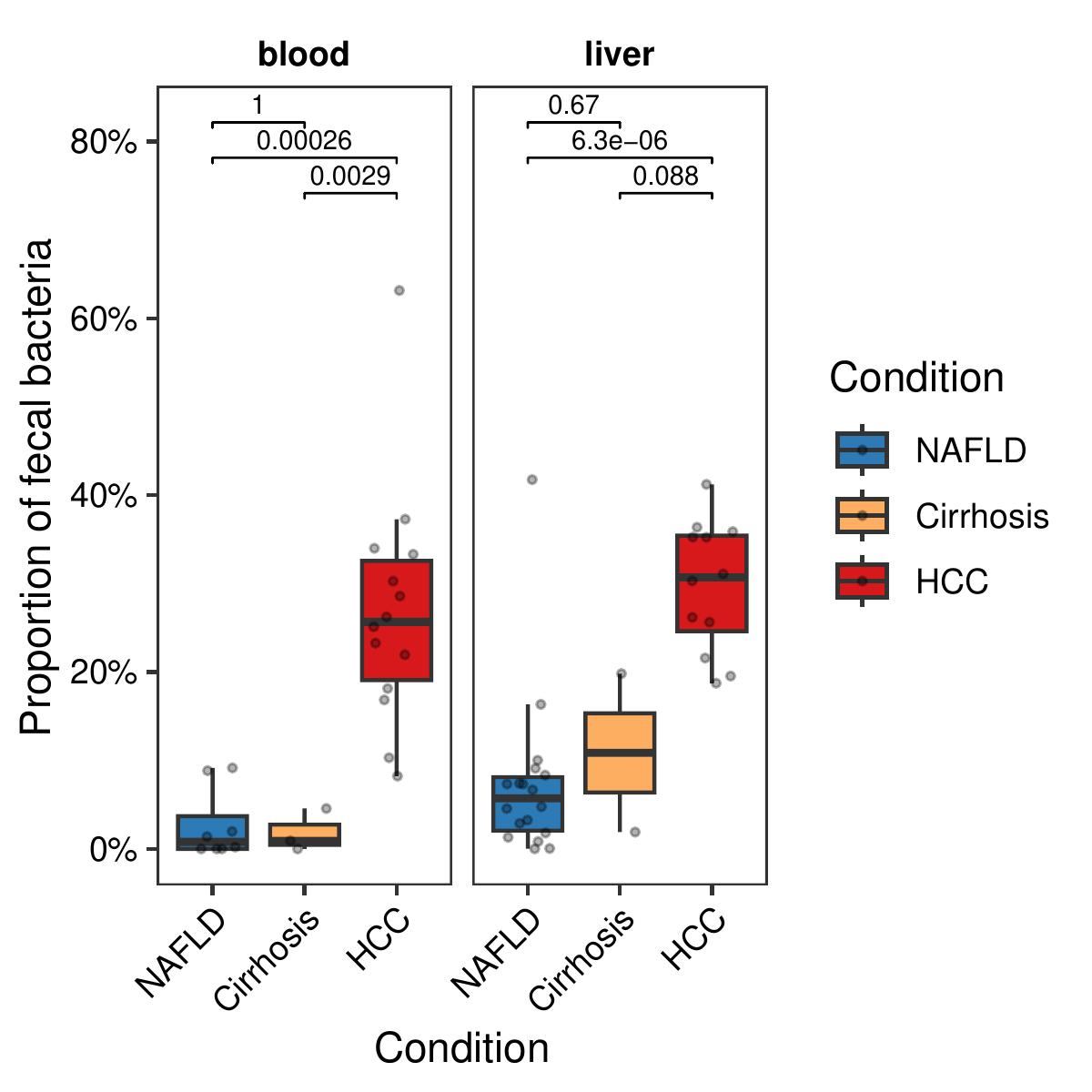


**Proportion of fecal bacteria in blood and liver 16S samples from patients with NAFLD as underlying condition.** The proportion of fecal bacteria is defined as the summed relative abundance of genera, which have a relative abundance of ≥ 0.1% in at least 5% of the fecal samples from the same disease condition group (NAFLD, cirrhosis, HCC). Statistical significance of pairwise group comparisons were assessed using the Mann-Whitney-U-test with NAFLD as reference.

**Supplementary Figure S5**


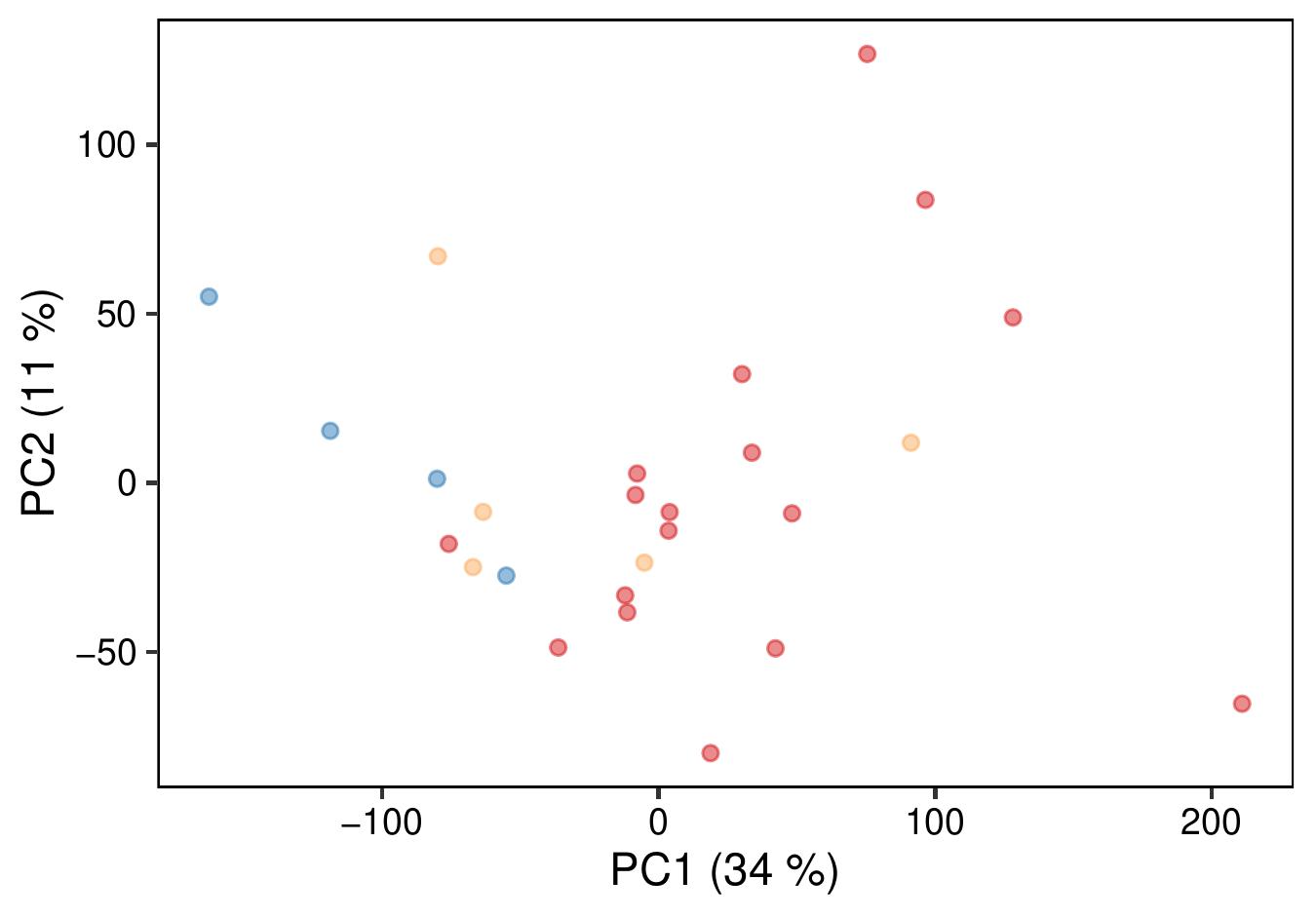


Principle component analysis of gene expression data quantified as transcripts per million (TPM). Color code: NAFLD (blue), cirrhosis (yellow), and HCC (red).

**Supplementary Figure S6**


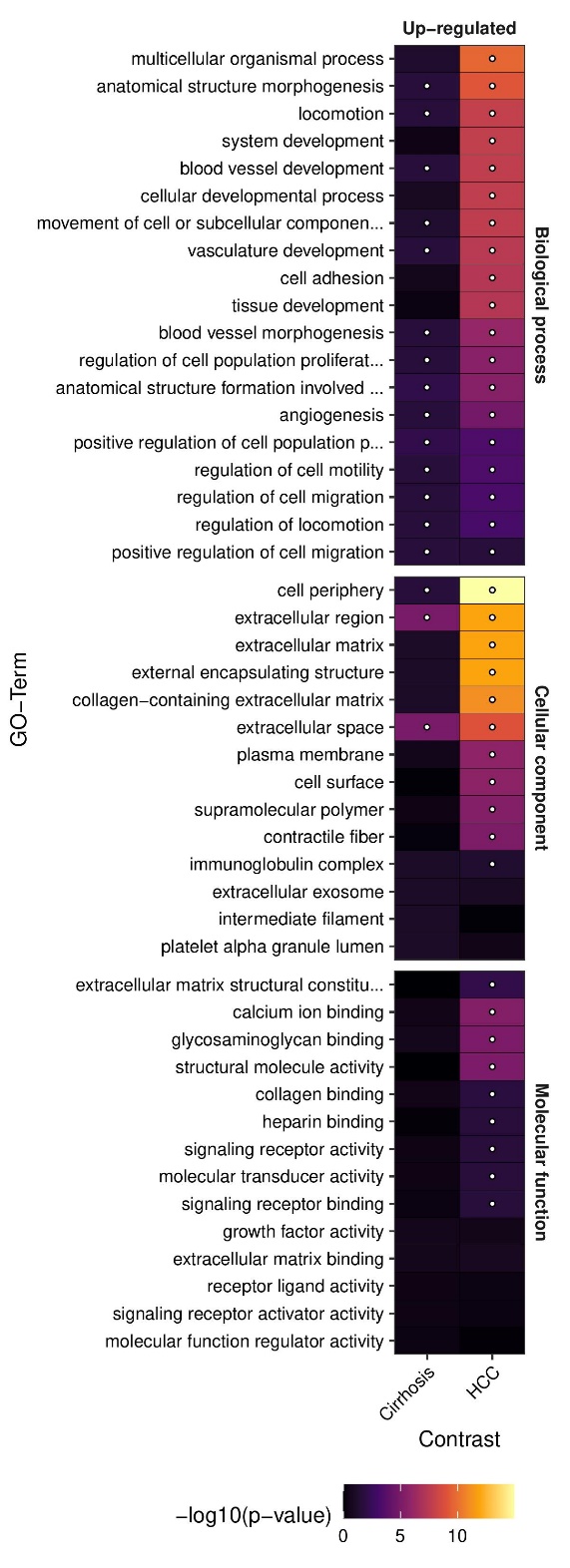


**Gene ontology (GO)-Term enrichment analysis results.** Up-regulated genes in cirrhosis and HCC in comparison to NAFLD were investigated for enrichment in specific GO-Terms of all three main categories: biological function, cellular component and molecular function. Statistically significant GO-Terms are marked with a white dot (Gene Set Enrichment Analysis using Fisher statistics, p < 0.05). No GO-Terms were enriched for down-regulated genes. Reported GO-Terms are limited to include at least the 10 most significant terms per GO-Term category and disease condition.

**References**

1. Marrero JA, Kulik LM, Sirlin CB, et al. Diagnosis, Staging, and Management of Hepatocellular Carcinoma: 2018 Practice Guidance by the American Association for the Study of Liver Diseases. *Hepatology*. 08 2018;68(2):723-750. doi:10.1002/hep.29913

2. easloffice@easloffice.eu EAftSotLEa, Liver EAftSot. EASL Clinical Practice Guidelines: Management of hepatocellular carcinoma. *J Hepatol*. 07 2018;69(1):182-236. doi:10.1016/j.jhep.2018.03.019

3. Tang A, Bashir MR, Corwin MT, et al. Evidence Supporting LI-RADS Major Features for CT- and MR Imaging-based Diagnosis of Hepatocellular Carcinoma: A Systematic Review. *Radiology*. 01 2018;286(1):29-48. doi:10.1148/radiol.2017170554

4. Chalasani N, Younossi Z, Lavine JE, et al. The diagnosis and management of nonalcoholic fatty liver disease: Practice guidance from the American Association for the Study of Liver Diseases. *Hepatology*. 01 2018;67(1):328-357. doi:10.1002/hep.29367

5. (EASL) EAftSotL, (EASD) EAftSoD, (EASO) EAftSoO. EASL-EASD-EASO Clinical Practice Guidelines for the management of non-alcoholic fatty liver disease. *J Hepatol*. 06 2016;64(6):1388-402. doi:10.1016/j.jhep.2015.11.004

6. Liver EAfSo, Higado ALpeEd. EASL-ALEH Clinical Practice Guidelines: Non-invasive tests for evaluation of liver disease severity and prognosis. *J Hepatol*. Jul 2015;63(1):237-64. doi:10.1016/j.jhep.2015.04.006

7. Kleiner DE, Brunt EM, Van Natta M, et al. Design and validation of a histological scoring system for nonalcoholic fatty liver disease. *Hepatology*. Jun 2005;41(6):1313-21. doi:10.1002/hep.20701

8. easloffice@easloffice.eu EAftSotLEa, Liver EAftSot. EASL Clinical Practice Guidelines for the management of patients with decompensated cirrhosis. *J Hepatol*. 08 2018;69(2):406-460. doi:10.1016/j.jhep.2018.03.024

9. Weissenborn K, Ennen JC, Schomerus H, Rückert N, Hecker H. Neuropsychological characterization of hepatic encephalopathy. *J Hepatol*. May 2001;34(5):768-73. doi:10.1016/s0168-8278(01)00026-5

10. Bale R, Widmann G, Schullian P, et al. Percutaneous stereotactic radiofrequency ablation of colorectal liver metastases. *Eur Radiol*. Apr 2012;22(4):930-7. doi:10.1007/s00330-011-2314-0

11. Bale R, Schullian P, Eberle G, et al. Stereotactic Radiofrequency Ablation of Hepatocellular Carcinoma: a Histopathological Study in Explanted Livers. *Hepatology*. 09 2019;70(3):840-850. doi:10.1002/hep.30406

12. Salter SJ, Cox MJ, Turek EM, et al. Reagent and laboratory contamination can critically impact sequence-based microbiome analyses. *BMC Biol*. Nov 2014;12:87. doi:10.1186/s12915-014-0087-z

13. Glassing A, Dowd SE, Galandiuk S, Davis B, Chiodini RJ. Inherent bacterial DNA contamination of extraction and sequencing reagents may affect interpretation of microbiota in low bacterial biomass samples. *Gut Pathog*. 2016;8:24. doi:10.1186/s13099-016-0103-7

14. Anhê FF, Jensen BAH, Varin TV, et al. Type 2 diabetes influences bacterial tissue compartmentalisation in human obesity. *Nat Metab*. Mar 2020;2(3):233-242. doi:10.1038/s42255-020-0178-9

15. Callahan BJ, McMurdie PJ, Rosen MJ, Han AW, Johnson AJ, Holmes SP. DADA2: High-resolution sample inference from Illumina amplicon data. *Nat Methods*. Jul 2016;13(7):581-3. doi:10.1038/nmeth.3869

16. Quast C, Pruesse E, Yilmaz P, et al. The SILVA ribosomal RNA gene database project: improved data processing and web-based tools. Article. *Nucleic Acids Research*. JAN 2013 2013;41(D1):D590-D596. doi:10.1093/nar/gks1219

17. Jari O, L. SG, Gouillaume BF, et al. vegan: Community Ecology Package

Ordination methods, diversity analysis and other functions for community and vegetation ecologists.

18. Bray JR, Curtis JT. An Ordination of the Upland Forest Communities of Southern Wisconsin. Ecological Monographs.

19. Pedro MA. pairwiseAdonis: Pairwise multilevel comparison using adonis. R package version 0.4. 2020.

20. Aysa K, Ludwig G, Marcel R, Levi W. lefser: R implementation of the LEfSE method for microbiome biomarker discovery, R package version 1.8.0. 2022.

21. Segata N, Izard J, Waldron L, et al. Metagenomic biomarker discovery and explanation. *Genome Biol*. Jun 24 2011;12(6):R60. doi:10.1186/gb-2011-12-6-r60

22. Schneider VA, Graves-Lindsay T, Howe K, et al. Evaluation of GRCh38 and de novo haploid genome assemblies demonstrates the enduring quality of the reference assembly. *Genome Res*. 05 2017;27(5):849-864. doi:10.1101/gr.213611.116

23. Ewels PA, Peltzer A, Fillinger S, et al. The nf-core framework for community-curated bioinformatics pipelines. *Nat Biotechnol*. 03 2020;38(3):276-278. doi:10.1038/s41587-020-0439-x

24. Dobin A, Davis CA, Schlesinger F, et al. STAR: ultrafast universal RNA-seq aligner. *Bioinformatics*. Jan 01 2013;29(1):15-21. doi:10.1093/bioinformatics/bts635

25. Patro R, Duggal G, Love MI, Irizarry RA, Kingsford C. Salmon provides fast and bias-aware quantification of transcript expression. *Nat Methods*. Apr 2017;14(4):417-419. doi:10.1038/nmeth.4197

26. Martin M. Cutadapt removes adapter sequences from high-throughput sequencing reads. EMBnet.journal2011.

27. Sayols S, Scherzinger D, Klein H. dupRadar: a Bioconductor package for the assessment of PCR artifacts in RNA-Seq data. *BMC Bioinformatics*. Oct 21 2016;17(1):428. doi:10.1186/s12859-016-1276-2

28. Wang L, Wang S, Li W. RSeQC: quality control of RNA-seq experiments. *Bioinformatics*. Aug 15 2012;28(16):2184-5. doi:10.1093/bioinformatics/bts356

29. Okonechnikov K, Conesa A, García-Alcalde F. Qualimap 2: advanced multi-sample quality control for high-throughput sequencing data. *Bioinformatics*. Jan 15 2016;32(2):292-4. doi:10.1093/bioinformatics/btv566

30. Daley T, Smith AD. Predicting the molecular complexity of sequencing libraries. *Nat Methods*. Apr 2013;10(4):325-7. doi:10.1038/nmeth.2375

31. Ewels P, Magnusson M, Lundin S, Käller M. MultiQC: summarize analysis results for multiple tools and samples in a single report. *Bioinformatics*. Oct 01 2016;32(19):3047-8. doi:10.1093/bioinformatics/btw354

32. Kurtzer GM, Sochat V, Bauer MW. Singularity: Scientific containers for mobility of compute. *PLoS One*. 2017;12(5):e0177459. doi:10.1371/journal.pone.0177459

33. da Veiga Leprevost F, Grüning BA, Alves Aflitos S, et al. BioContainers: an open-source and community-driven framework for software standardization. *Bioinformatics*. Aug 15 2017;33(16):2580-2582. doi:10.1093/bioinformatics/btx192

34. Gu Z, Eils R, Schlesner M. Complex heatmaps reveal patterns and correlations in multidimensional genomic data. *Bioinformatics*. 09 15 2016;32(18):2847-9. doi:10.1093/bioinformatics/btw313

35. Adrian A, Rahnenfuhrer J. Enrichment Analysis for Gene Ontology. doi:DOI:10.18129/B9.bioc.topGO
